# Upper Parietal Meningioma Showing Foster Kennedy Syndrome
*A case shown at the Clinical Meeting of the Society on Wednesday, December 11th, 1946.


**Published:** 1947

**Authors:** Diana J. Kinloch Beck

**Affiliations:** Honorary Neurosurgeon, Royal Free Hospital, Elizabeth Garrett Anderson Hospital and Marie Curie Hospital, London; Regional Adviser in Neurosurgery to the South-West Region, Ministry of Health


### UPPER PARIETAL MENINGIOMA SHOWING FOSTER KENNEDY SYNDROME.*

BY

DIANA J. KINLOCH BECK, M.B., B.S., F.R.C.S., F.R.C.S.E.

*Honorary Neurosurgeon, Royal Free Hospital, Elizabeth Garrett Anderson Hospital and Marie Curie Hospital, London ; Regional Adviser in Neurosurgery to the South-West Region, Ministry of Health.*

### TOTAL EXCISION

R.P., a draughtsman, aged 39. During the twelve years before admission, the patient had eight right-sided Jacksonian attacks, which were preceded by a feeling of spatial disorientation. They occasionally lasted for hours, and were then terminated by unconsciousness when he had reached a condition of exhaustion. In addition to the Jacksonian attacks he experienced bouts of severe, and at times unbearable, cramp-like pain in the right limbs. Throughout the illness there was increasing difficulty in using the right hand and also in appreciating textures with it. For one year he had suffered increasingly severe frontal headache and progressive deterioration of vision.

He was admitted to Neurosurgical Unit on 14th April, 1946. An intelligent, highly-strung man, left-handed, but writes with right hand ; speech functions normal, no anosmia ; visual acuity, R. J.1. ; L. J.6 : visual fields full ; fundi : R. papilloedema 2–3 D., L. optic atrophy with clearly defined margins. Motor functions normal, except for diminished facility with right hand. Marked astereognosis of right hand ; diminished 2-point discrimination, and diminished appreciation of texture in right hand and right foot : equivocal right plantar response.

X-rays of skull show fairly extensive area of pitting in left upper parietal region and sun-ray appearance in same area ; large diploic vessels in left upper parietal region and erosion of posterior clinoid processes. Cerebro-spinal fluid : pressure 340 mm., protein 90 mgm. per cent. Diagnosis : Left upper parietal meningioma showing Foster Kennedy Syndrome. Ventriculography on 19th April, 1946, confirmed a left upper parietal space-occupying lesion.

Operation on 19th April, 1946, by Miss Beck. Left lateral osteo-plastic flap : complete removal of upper left parietal meningioma (6·5 × 6·5 × 7 cm.) attached to dura over an area 2·5 cm. in diameter. Pathological report : An endothelial meningioma. No invasion of bone by tumour.

* A case shown at the Clinical Meeting of the Society on Wednesday, December 11th, 1946.

The patient was discharged walking from hospital twenty-one days after operation: right optic disc then flat, visual acuity, R. and L., J.1. He has been working full-time as a draughtsman since the beginning of October, 1946. He has had no attacks and no headaches since operation. Vision J.1 on each side, right disc normal; left disc paler than right; there is slight clumsiness in handling objects with right hand, but no loss of power; still some astereognosis and some difficulty in appreciation of two points and of textures with right hand (much less than before operation).

The interest of this case centres round the Foster Kennedy syndrome, which is marked by optic atrophy on one side and papilloedema on the other. Leslie Paton's[1] name should be added to the eponym, for in 1909 (two years before Kennedy's paper[2] was published) he first described "a marked primary atrophy in the left eye with an intense neuritis in the right" in a patient subsequently found to have "a large frontal tumour excavating the under surface of both frontal lobes but more on the left side than on the right." The syndrome is recognized as a diagnostic criterion of the existence of an expanding lesion of the baso-frontal region or anterior fossa, and although Kennedy stated that it could not be simulated by any lesion failing to exert pressure on the inferior surface of one or other frontal lobe, it has been described in many intracranial conditions of very varying pathology. The absence of anosmia and the patient's normal mentality in the present case made the common causative lesions, an olfactory groove meningioma or a tumour of the frontal lobe, most unlikely. The long history of Jacksonian attacks with disturbances of the discriminative aspects of sensation led to a clinical diagnosis of a left upper parietal meningioma: the raised cerebro-spinal fluid protein and the plain X-rays supported this. Uncertainty as to the exact significance of the Foster Kennedy syndrome indicated the need for ventriculography.

This case illustrates the need to interpret the syndrome in the light of other signs and to pay attention to the stage at which the eye signs develop. Their late appearance, as in the present case, means that they are the result of a rise of intracranial pressure. On this premise, it may seem difficult to account for the appearance of the disc on the side of the lesion, which conforms to the description of primary optic atrophy. It is known that papilloedema may differ greatly in intensity in the two eyes, and it is not impossible that with a slowly growing tumour, papilloedema may develop on the ipsi-lateral side and be succeeded by optic atrophy, which although actually consecutive, is indistinguishable from that usually regarded as being of the primary variety.

REFERENCES.

[1] Paton, Leslie (1909), *Brain*, xxxii. 65.
[2] Kennedy, Foster (1911), *Am.J.M.Sc.*, cxlii. 355.

